# Sinus pneumatisation and Keros type as predictors of anterior ethmoidal artery configuration: a 3D-reconstructed computed tomography analysis

**DOI:** 10.1017/S0022215126104290

**Published:** 2026-03

**Authors:** Yeon Hee Im, Yujeong Hong, Dong Jun Kim, Beom Cho Jun

**Affiliations:** 1 Department of Otorhinolaryngology-Head and Neck Surgery, St. Vincent’s Hospital, College of Medicine, The Catholic University of Korea, Seoul, Republic of Korea; 2 Department of Otorhinolaryngology-Head and Neck Surgery, Uijeongbu St. Mary’s Hospital, College of Medicine, The Catholic University of Korea, Seoul, Republic of Korea.

**Keywords:** anatomic variation, ethmoid sinus, frontal sinus, paranasal sinuses, skull base, tomography, X-ray computed

## Abstract

**Objective:**

This study aimed to examine anatomical factors predicting anterior ethmoidal artery type.

**Methods:**

Paranasal sinus computed tomography images from adult patients were reviewed. Anterior ethmoidal artery types were categorised based on skull base relationship: type A (embedded within skull base), type B (in a protruding canal) and type C (separated from skull base). Statistical analyses encompassed inter-type comparisons, multinomial logistic regression and correlation analysis.

**Results:**

Anterior ethmoidal artery types differed significantly in lateral lamella height and Keros classification, with type C showing the greatest lateral lamella height and predominant Keros III. Type C was additionally characterised by enlarged frontal sinus volume, increased volume and height of frontal recess anterior cells and higher supraorbital ethmoid cell prevalence. The anterior ethmoidal artery–skull base distance demonstrated significantly positive correlations with lateral lamella height, frontal sinus volume and the volume and height of frontal recess anterior cells.

**Conclusion:**

More extensive pneumatisation of frontal sinus and ethmoid cells and increased lateral lamella height might predict a free-running anterior ethmoidal artery.

## Introduction

The anterior ethmoidal artery (AEA) is a critical anatomical structure in paranasal sinus surgery. Intra-operative injury can lead to heavy bleeding, orbital haematoma, visual compromise, cerebrospinal fluid leakage, and, rarely, intracranial haemorrhage, emphasising the need for precise identification and preservation of the vessel.^1^ Moreover, during an external rhinotomy for sinonasal tumour resection, locating the lacrimal crest and subsequently tracing the AEA along the frontoethmoidal suture line aids in delineating the skull base.^2^ The AEA is likewise used as a practical landmark to orient the frontal recess and frontal sinus or the anterior skull base during endoscopic sinus surgery, and in transcribriform approaches, it is typically identified and ligated preemptively.^3^

The artery’s relationship to the skull base, however, varies across individuals along its course from the orbit to the cribriform plate. In this context, three types are commonly described: type A, in which the artery runs within or immediately adjacent to the skull base; type B, in which it passes through a canal that protrudes below the skull base; and type C, in which it courses freely within the ethmoid sinus, separated from the skull base.^4^ The configuration may also be asymmetric, with different types present on the right and left sides in the same patient.^5^ Because the type C artery is suspended away from the skull base and exposed within the ethmoid cavity, it carries a higher risk of intra-operative injury and thus demands particular caution.^3^

Pre-operative identification of the AEA type and its course on imaging is thus important to reduce the risk of vascular injury and to facilitate a safer operation. Particularly paranasal sinus (PNS) computed tomography (CT), which renders bony anatomy with high fidelity, allows estimation of the AEA pathway on coronal images using two landmarks—the notch of the medial orbital wall and the anterior ethmoidal groove on the lateral lamella of the cribriform plate.^6^^,^^7^ Nevertheless, delineation can be challenging when prior surgery distorted the anatomy, when the bony wall of the anterior ethmoidal canal is dehiscent or markedly thinned or when a mucocele, extensive polyposis or a tumour involving ethmoid sinus is present.^3^^,^^8^ Therefore, this study aimed to identify factors that predict the AEA type and position. Using 3D reconstruction of PNS CT, we quantified frontal and ethmoidal pneumatisation and examined their associations with AEA type and its distance from the skull base.

## Materials and methods

### Patients and study design

This study retrospectively analysed the PNS CT images of the patients. The study received approval from the Institutional Review Board of Uijeongbu St. Mary’s Hospital, the Catholic University of Korea, in 2024 (approval number UC24RISI0119), with the exemption of patient consent. Data of patients who have undertaken PNS CT between November and December of 2023 were initially extracted. Patients under 18 years of age, those with craniofacial anomaly, those with history of nose surgery or traumatic facial fractures and those with abnormal lesions in the sinonasal region detected in PNS CT were excluded.

**Figure 1.**
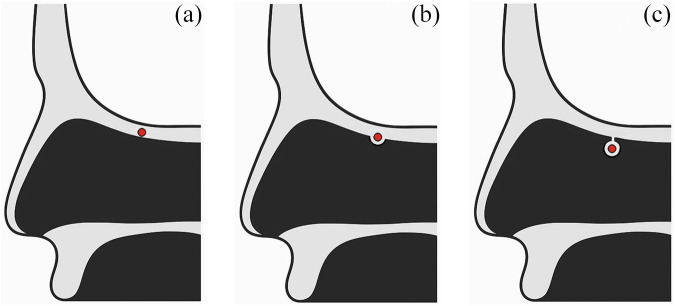
Classification of anterior ethmoidal artery types according to their relationship to the skull base. (a) Type A: anterior ethmoidal artery buried in the skull base. (b) Type B: protruding equal to or greater than 1 mm from the skull base. (c) Type C: located in the ethmoid sinus separately from the skull base.

Patients were categorised into three types according to the location of AEA in PNS CT (type A: AEA buried in the skull base; type B: protruding equal to or more than 1 mm from the skull base; type C: AEA located in the ethmoid sinus separately from the skull base) (Figure 1).^4^^,^^9^ Clinical characteristics were compared among the three types. Multinomial logistic regression analysis was performed to adjust for potential confounders and determine independent factors affecting AEA location. Correlation analysis and partial correlation analysis were additionally utilised to identify the association between the AEA-skull base distance (D_AS_) and the other anatomical variables as well as between the height of cribriform plate lateral lamella (H_LL_) and the other variables.

### Measurement of length, area and volume parameters

**Figure 2.**
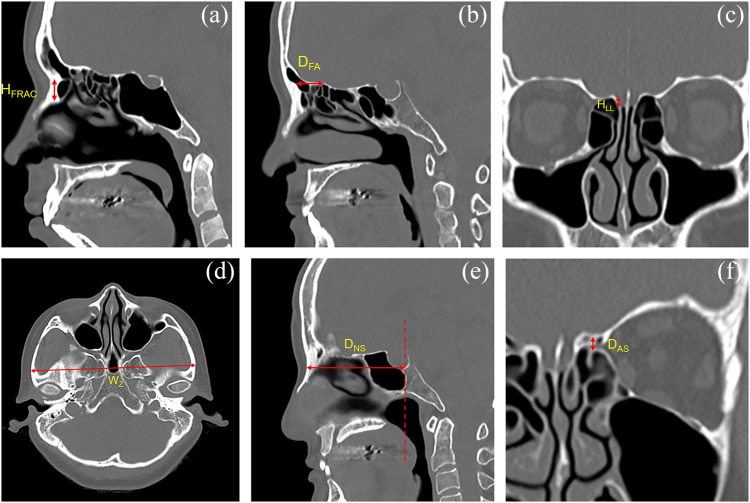
Linear measurements on paranasal sinus computed tomography (CT). (a) Height of the frontal recess anterior cells (H_FRAC_). (b) Frontal beak–anterior ethmoidal artery distance (D_FA_). (c) Height of the cribriform plate lateral lamella (H_LL_). (d) Interzygomatic distance (W_Z_). (e) Nasion–sphenoid distance (D_NS_). (f) Anterior ethmoidal artery–skull base distance (D_AS_).

When performing PNS CT, patients were positioned supine, and the angle of the hard palate was adjusted to be perpendicular to the scanning table of the CT device. Images were acquired with a 1-mm slice thickness. The frontal recess anterior cells (FRAC)—the anteriorly located cells within the frontal recess including the Agger nasi and supra-Agger cells per the International Frontal Sinus Anatomy Classification—were identified, and their height (H_FRAC_) was defined as the vertical distance from the lowest to the highest point (Figure 2A).^10^ The frontal beak–AEA distance (D_FA_) was measured on the sagittal view containing the entry point of the AEA into the skull base, as the horizontal distance between the frontal beak and the AEA (Figure 2B). H_LL_ was determined on coronal images at the point where the AEA entered the skull base and was defined as the vertical height of the lateral lamella; this value was then used to classify the Keros type (type I: < 4 mm; type II: ≥ 4 and < 8 mm; type III: ≥ 8 mm) (Figure 2C).^11^ The interzygomatic distance (W_Z_) was defined on the axial view at the highest level where the zygomatic arch meets the temporal bone, as the greatest distance between the right and left zygomas (Figure 2D). In addition, the nasion–sphenoid distance (D_NS_) was designated on the sagittal view including the nasion, as the horizontal distance from the anterior border of the nasal bone to the posterior-most point of sphenoid sinus posterior wall (Figure 2E). The D_AS_ was defined as the vertical distance from the floor of the AEA canal at its midpoint to the inferior surface of the skull base at the corresponding site (Figure 2F).

**Figure 3.**
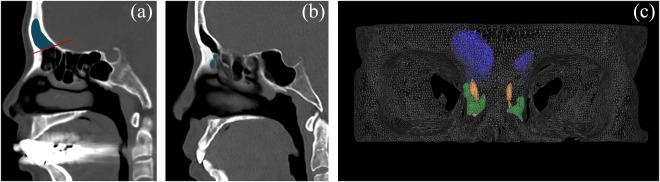
Volumetric measurements of frontal sinus and frontal recess anterior cells (FRAC) on paranasal sinus computed tomography (CT). (a) On each sagittal slice of paranasal sinus CT, a reference line (red line) was drawn from the frontal ridge to the point where the septum separating the frontal and ethmoid sinuses meets the skull base, serving as the inferior boundary of frontal sinus (blue shading). (b) Sagittal slice illustrating the FRAC (blue shading). (c) 3D reconstruction of frontal sinus volume (V_FS_), the volume of FRAC (V_FRAC_) and the frontal recess passage; frontal sinuses are shown in blue, FRAC in green and frontal recess passage in yellow.

Volumes of 3D parameters were measured using V-works software (Version 4.0, Cybermed Inc., Seoul, South Korea). Surface renderings were generated using −318 Hounsfield units (HU) as the air–density threshold. To measure the frontal sinus volume (V_FS_), reference lines were drawn on PNS CT sagittal views containing the frontal recess, extending from the frontal ridge to the point where the septum separating the frontal and ethmoid sinuses meets the skull base; these lines were designated as the inferior boundary for the calculation of frontal sinus volume (Figure 3A). The volume of frontal recess anterior cells (V_FRAC_) was defined as the sum of the volumes of the Agger nasi cell and supra-Agger cells located in the anterior portion of the frontal recess (Figure 3B, 3C).

### Statistical analysis

All statistical analyses were carried out using IBM SPSS Statistics (version 28; IBM Corp., Armonk, NY, USA). Continuous data were expressed as mean values with corresponding standard deviations, whereas categorical data were summarised as frequencies and percentages. Comparisons of continuous variables across multiple groups were conducted using one-way analysis of variance followed by Tukey’s post hoc test or the Kruskal–Wallis test when appropriate. For categorical variables, group differences were assessed with Pearson’s χ^2^ test or Fisher’s exact test, applying Bonferroni correction for multiple comparisons. To adjust for potential confounders and to determine independent predictors of ternary outcomes, multinomial logistic regression models were employed. Correlations between continuous variables were examined using Spearman’s correlation analysis, and partial Spearman’s correlation was further applied to account for additional confounding variables. A two-tailed *p* value less than 0.05 was considered indicative of statistical significance.

## Results and analysis

A total of 56 participants (112 nasal cavities) were finally included in this study. Average age was 45.1 ± 17.7 years, and of the total participants 29 (51.8 %) patients were male and 27 (48.2 %) were female.

Table 1 summarises the comparison of clinical characteristics among types A, B and C. A significant difference was observed in the distribution of sex across the three types, with the proportion of males being highest in type C and lowest in type A, showing a significant difference between types A and C (27.8 % vs. 64.6 %; *p* = 0.005). No significant difference was found in age. Both V_FS_ and V_FRAC_ demonstrated significant differences, with type C showing significantly greater values compared with types A and B (V_FS_: 1.66 ± 1.18 vs. 2.01 ± 2.11 vs. 4.34 ± 3.21 cm^3^; *p* < 0.001 for both; V_FRAC_: 108.40 ± 74.78 vs. 95.82 ± 66.23 vs. 189.59 ± 135.40 mm^3^; *p* = 0.003 and <0.001, respectively). H_FRAC_ was also highest in type C, and a significant difference was noted between types B and C (1.20 ± 0.42 vs. 1.48 ± 0.51 cm; *p* = 0.030). In contrast, D_FA_ did not differ significantly among the three types. The prevalence of supraorbital ethmoid cells (SOEC) was highest in type C, with a significant difference between types A and C (11.1 % vs. 41.5 %; *p* = 0.017). H_LL_ and Keros type also revealed significant differences. H_LL_ was significantly greater in type C than in types A and B (4.11 ± 1.61 vs. 4.16 ± 1.27 vs. 6.52 ± 2.79 mm; *p* < 0.001). The proportions of Keros type I and II were lowest in AEA type C, while Keros type III was most frequent in AEA type C, showing a significant difference compared with AEA types A and B. The presence of frontal cells did not differ significantly among the types. Likewise, W_Z_ and the D_NS_ showed no significant inter-type differences.

**Table 1.** Comparison of clinical findings among the AEA types

Parameter	Total (*n* = 112)	AEA type A (*n* = 18)	AEA type B (*n* = 29)	AEA type C (*n* = 65)	*p* value
Male, *n* (%)	58 (51.8%)	5 (27.8%)^‡^	11 (37.9%)	42 (64.6%)^‡^	0.005
Age	45.1 ± 17.7	40.0 ± 16.4	46.1 ± 17.3	46.0 ± 18.2	0.416
V_FS_, cm^3^	3.30 ± 2.96	1.66 ± 1.18^‡^	2.01 ± 2.11^†^	4.34 ± 3.21^†^^,^^‡^	<0.001
V_FRAC_, mm^3^	152.26 ± 120.39	108.40 ± 74.78^‡^	95.82 ± 66.23^†^	189.59 ± 135.40^†^^,^^‡^	<0.001
H_FRAC_, cm	1.38 ± 0.49	1.30 ± 0.47	1.20 ± 0.42^†^	1.48 ± 0.51^†^	0.030
D_FA_, cm	1.60 ± 0.30	1.61 ± 0.32	1.67 ± 0.28	1.57 ± 0.30	0.183
SOEC, *n* (%)	34 (30.4%)	2 (11.1%)^‡^	5 (17.2%)	27 (41.5%)^‡^	0.009
H_LL_, mm	5.52 ± 2.79	4.11 ± 1.61^‡^	4.16 ± 1.27^†^	6.52 ± 2.79^†^^,^^‡^	<0.001
^a^Keros type, *n* (%)					<0.001
Type I	30 (26.8%)	6 (33.3%)^‡^	13 (44.8%)^†^	11 (16.9%)^†^^,^^‡^	
Type II	61 (54.5%)	12 (66.7%)^‡^	16 (55.2%)^†^	33 (50.8%)^†^^,^^‡^	
Type III	21 (18.8%)	0 (0.0%)^‡^	0 (0.0%)^†^	21 (32.3%)^†^^,^^‡^	
Presence of frontal cell	55 (49.1%)	11 (61.1%)	10 (34.5%)	34 (52.3%)	0.151
W_Z_, cm	12.69 ± 0.70	12.49 ± 0.64	12.64 ± 0.54	12.77 ± 0.77	0.289
D_NS_, cm	7.30 ± 0.63	7.14 ± 0.54	7.43 ± 0.70	7.30 ± 0.63	0.083

AEA = anterior ethmoidal artery; D_FA_ = frontal beak–AEA distance; D_NS_ = nasion–sphenoid distance; H_FRAC_ = height of frontal recess anterior cells, H_LL_ = lateral lamella height; SOEC = supraorbital ethmoid cell; V_FRAC_ = volume of frontal recess anterior cells; V_FS_ = frontal sinus volume; W_Z_ = interzygomatic distance;

a Keros type: type I, a depth of the olfactory fossa < 4 mm; type II, a depth ≥ 4 and < 8 mm; type III, a depth ≥ 8 mm.

* Significant difference between types A vs. B.

† Significant difference between types B vs. C.

‡ Significant difference between types A vs. C.

The results of the multinomial logistic regression analysis, performed to control for potential confounding effects and to evaluate the independent influence of each CT parameter on AEA type, are presented in Table 2. Variables that demonstrated significant differences in the initial inter-type comparisons were included as independent variables. In the comparison between type A and type B the lack of significant associations between the CT parameters and AEA types remained unchanged after adjustment for confounders. In contrast, in the comparison between type B and type C, V_FS_, V_FRAC_ and H_LL_ remained significantly different, and higher values of each variable was associated with increased probability of belonging to type C (V_FS_: odds ratio [OR] 1.343, 95 % confidence interval [CI] 1.006–1.793; *p* = 0.045; V_FRAC_: OR 1.010, 95 % CI 1.001–1.019; *p* = 0.028; H_LL_: OR 1.877, 95 % CI 1.261–2.794; *p* = 0.002), whereas H_FRAC_ lost its significance. Furthermore, in the comparison between type A and type C, H_LL_ continued to show a significant association (OR 2.020; 95 % CI 1.270–3.212; *p* = 0.003), while sex, V_FS_, V_FRAC_ and presence of SOEC were no longer significantly related to AEA types.

**Table 2.** Multinomial logistic regression analysis for identifying independent factors associated with AEA type

Parameter		AEA type A vs. B	AEA type B vs. C	AEA type A vs. C
Male, *n* (%)	OR [95% CI]^a^	1.756 [0.440−7.015]	1.288 [0.389−4.275]	2.263 [0.528−9.694]
	*p* value	0.425	0.679	0.271
V_FS_, cm^3^	OR [95% CI]^a^	1.068 [0.702−1.626]	1.343 [1.006−1.793]	1.435 [0.964−2.135]
	*p* value	0.758	0.045*	0.075
V_FRAC_, mm^3^	OR [95% CI]^a^	0.998 [0.998−1.008]	1.010 [1.001−1.019]	1.008 [0.999−1.018]
	*p* value	0.735	0.028*	0.090
H_FRAC_, cm	OR [95% CI]^a^	0.428 [0.077−2.391]	0.986 [0.232−4.196]	0.423 [0.077−2.333]
	*p* value	0.334	0.985	0.323
SOEC, *n* (%)	OR [95% CI]^a^	2.513 [0.305−20.833]	1.631 [0.365−7.299]	4.098 [0.523−32.258]
	*p* value	0.392	0.522	0.179
H_LL_, mm	OR [95% CI]^a^	1.076 [0.701 – 1.652]	1.877 [1.261−2.794]	2.020 [1.270−3.212]
	*p* value	0.737	0.002*	0.003*

AEA = anterior ethmoidal artery; CI = confidence interval; H_FRAC_ = height of frontal recess anterior cells; H_LL_ = lateral lamella height; OR = odds ratio; SOEC = supraorbital ethmoid cell; V_FRAC_ = volume of frontal recess anterior cells; V_FS_ = frontal sinus volume.

a Continuous variables: odds ratio of belonging to the latter group per 1-unit increase. Categorical variables: odds ratio of belonging to the latter group for each category (e.g., male sex or presence of a supraorbital cell).

* *p* < 0.05.

The results of the correlation analysis between D_AS_ values and the other continuous variables are presented in Table 3. Age and D_FA_ showed no significant associations with D_AS_ values. In contrast, V_FS_, V_FRAC_, H_FRAC_ and H_LL_ each demonstrated a significant positive correlation with D_AS_ (V_FS_: Spearman correlation coefficient [r_s_] 0.401, *p* < 0.001; V_FRAC_: r_s_ 0.436, *p* < 0.001; H_FRAC_: r_s_ 0.269, *p* = 0.004; H_LL_: r_s_ 0.604, *p* < 0.001). W_Z_ and D_NS_ were also positively correlated with D_AS_ (W_z_: r_s_ 0.219, *p* = 0.020; D_NS_: r_s_ 0.226, *p* = 0.016). Furthermore, in the partial correlation analysis controlling for the effects of age, W_Z_ and D_NS_, the positive correlations between D_AS_ and V_FS_, V_FRAC_, H_FRAC_ and H_LL_ remained significant respectively (V_FS_: r_s_ 0.365, *p* < 0.001; V_FRAC_: r_s_ 0.460, *p* < 0.001; H_FRAC_: r_s_ 0.257, *p* = 0.007; H_LL_: r_s_ 0.612, *p* < 0.001).

**Table 3.** Correlation analysis between AEA–skull base distance and the other continuous variables

Parameter		Age	V_FS_	V_FRAC_	H_FRAC_	D_FA_	H_LL_	W_Z_	D_NS_
Correlation analysis	r_s_	0.166	0.401	0.436	0.269	−0.065	0.604	0.219	0.226
	*p* value	0.079	<0.001*	<0.001*	0.004*	0.494	<0.001*	0.020*	0.016*
Partial correlation analysis controlling age, W_Z_ and D_NS_	r_s_		0.365	0.460	0.257	−0.085	0.612		
	*p* value		<0.001*	<0.001*	0.007*	0.380	<0.001*		

AEA = anterior ethmoidal artery; V_FS_ = frontal sinus volume; V_FRAC_ = volume of frontal recess anterior cells; H_FRAC_ = height of frontal recess anterior cells; D_FA_ = frontal beak–AEA distance; H_LL_ = lateral lamella height; W_Z_ = interzygomatic distance; D_NS_ = nasion–sphenoid distance; *r_s_* = Spearman correlation coefficient.

* *p* < 0.05.

The correlation analyses between the H_LL_ and other continuous variables are summarised in Table 4. H_LL_ was not significantly associated with age, D_FA_, W_Z_ and D_NS_. In contrast, H_LL_ demonstrated significant positive correlations with V_FS_, V_FRAC_, H_FRAC_ and D_AS_ (V_FS_: r_s_ 0.285, *p* = 0.002; V_FRAC_: r_s_ 0.264, *p* = 0.005; H_FRAC_: r_s_ 0.218, *p* = 0.021; D_AS_: r_s_ 0.604, *p* < 0.001).

**Table 4.** Correlation analysis between height of cribriform plate lateral lamella and the other continuous variables

Parameter		Age	V_FS_	V_FRAC_	H_FRAC_	D_FA_	D_AS_	W_Z_	D_NS_
Correlation analysis	r_s_	−0.059	0.285	0.264	0.218	−0.137	0.604	0.136	0.181
	*p* value	0.539	0.002*	0.005*	0.021*	0.149	<0.001*	0.152	0.056

D_AS_ = anterior ethmoidal artery–skull base distance; D_NS_ = nasion–sphenoid distance; D_FA_ = frontal beak–anterior ethmoidal artery distance; H_FRAC_ = height of frontal recess anterior cells; *r_s_* = Spearman correlation coefficient; V_FRAC_ = volume of frontal recess anterior cells; V_FS_ = frontal sinus volume; W_Z_ = interzygomatic distance;

* *p* < 0.05.

## Discussion

In endoscopic sinus surgery, AEA is a critical surgical landmark. Anatomically, the AEA branches from the ophthalmic artery, itself a division of the internal carotid artery. Within the orbit, it courses between the superior oblique and medial rectus muscles before traversing the anterior ethmoidal foramen of the medial orbital wall. It then proceeds through the anterior ethmoidal canal along the ethmoid sinus or ethmoid roof, typically running obliquely in an anteromedial direction, and enters the anterior cranial fossa via the lateral lamella of the cribriform plate. The AEA supplies the frontal and ethmoid sinuses, the nasal roof and septum and the meninges of the anterior cranial fossa.^1^^,^^12^^,^^13^ The AEA is usually grouped into three categories by its position relative to the skull base, with substantial variation among patients.^4^ However, data are still limited on which clinical features are associated with each type.

In our cohort, the distribution of AEA types was 16.1 % for type A, 25.9 % for type B and 58.0 % for type C. This profile contrasts with earlier reports in which the artery was most frequently embedded within the skull base.^4^^,^^12^^,^^14^^–^^16^ However, studies mirroring our findings—showing a predominance of type C—have also been published.^17^^–^^19^ A recent meta-analysis further noted a type C configuration in 43.09 %, reporting that the frequencies of each AEA phenotype varied considerably across studies.^13^ Such heterogeneity likely reflects differences in population characteristics, including regional and ethnic factors, as well as imaging protocols and classification criteria.

Sağlam *et al*. and Poteet *et al*. each reported that AEA type C was significantly more common in men than in women and that male patients tended to have a greater lateral lamella height.^5^^,^^20^ In contrast, other investigations found no significant sex difference in the AEA–skull base distance.^1^^,^^3^ Gibelli *et al*. and Randhawa *et al*. also did not observe sex-based differences in the distribution of AEA types.^21^^,^^22^ In our analysis, the proportion of men was significantly higher in type C than in type A on unadjusted comparison; however, this association lost significance after adjusting for potential confounders (V_FS_, V_FRAC_, H_FRAC_, SOEC and H_LL_). Because these covariates reflect paranasal sinus volumes and degrees of pneumatisation, the attenuation of the sex effect after adjustment of the confounders might be explained by the general tendency for men to exhibit greater sinus pneumatisation and larger sinus volumes.^23^^,^^24^

Several studies have linked craniofacial anatomy to AEA position like our study. Higher lateral lamella height or the presence of Keros type III has been associated with an increased likelihood that the AEA courses apart from the skull base in the previous studies.^6^^,^^14^^,^^16^^,^^20^^,^^25^ In an Indian cohort analysed by Randhawa *et al*., Keros type III was not observed, yet the proportion of Keros type II rose progressively from AEA type A to B to C (72.6 %, 83.5 % and 94.2 %, respectively).^22^ Other authors have reported that a longer anteroposterior extent of the lateral lamella similarly favors a more inferiorly positioned AEA.^26^ In line with these observations, our data showed that AEA type is significantly associated with both H_LL_ and Keros classification. Type C exhibited a significantly higher H_LL_ than types A and B. With respect to Keros classification, AEA type C had significantly lower proportions of Keros types I and II and a higher proportion of type III. Notably, Keros type III occurred exclusively in AEA type C in our cohort. These differences in H_LL_ remained significant in multinomial logistic regression. Moreover, correlation analysis demonstrated a significant positive relationship between the D_AS_ and H_LL_, which persisted after adjustment for age as well as W_Z_ and D_NS_ reflecting head size to account for potential confounding.

Several previous investigations have examined the relationship between the presence of a SOEC and AEA position. Multiple studies have reported that patients with an SOEC have significantly higher odds of belonging to AEA type C than those without an SOEC.^5^^,^^14^^,^^27^ Another study found that greater expansile development of the SOEC was associated with a significantly increased distance between the AEA and the skull base.^28^ Consistent with these observations, the prevalence of SOEC in our cohort rose progressively from AEA type A to B to C, with a significant difference between types A and C, even though this association weakened and lost statistical significance after adjustment for potential confounders. In contrast to SOEC, the presence of a suprabullar cell has been reported to show no significant association with AEA position.^14^ El-Anwar *et al*. further observed that supraorbital pneumatisation is associated with increases not only in the AEA–skull base distance but also in the AEA–frontal sinus ostium distance and in the angle between the AEA and the lamina papyracea.^3^ Another study, however, has reported no significant relationship between supraorbital pneumatisation and either AEA grade or Keros type.^22^ Moreover, one study concluded that the AEA’s location relative to the basal lamella (anterior, within or posterior) is not significantly associated with AEA type.^27^

In this study, V_FS_, V_FRAC_ and H_FRAC_ differed significantly according to AEA type. In multinomial logistic regression analysis, the differences in V_FS_ and V_FRAC_ between the types B and C remained significant, whereas H_FRAC_ fell below statistical significance. Correlation analyses also showed positive associations between D_AS_ and each of V_FS_, V_FRAC_ and H_FRAC_, which persisted after adjustment for the factors related to head size. Although Çomoğlu *et al*. did not measure frontal sinus volume, they stratified frontal sinus pneumatisation above the orbital roof into three grades and found that more extensive pneumatisation was linked to higher H_LL_ and a greater likelihood of the AEA coursing away from the skull base.^29^

In this study, H_LL_ also demonstrated significant positive correlations with V_FS_, V_FRAC_ and H_FRAC_. This finding suggests that more extensive pneumatisation of frontal sinus and anterior ethmoid cells tends to accompany a higher H_LL_. Consistent with this interpretation, Gumus and Yildirim reported that patients with hyperpneumatised frontal sinuses exhibited significantly higher H_LL_ than those without hyperpneumatisation.^30^ Moreover, in our dataset, V_RS_, V_FRAC_, H_FRAC_, presence of SOEC, H_LL_ and Keros type were each associated with AEA type and/or D_AS_. These observations indicate that individuals with more pneumatisation of the anterior ethmoid cells are more likely to have AEA positioned separately from the skull base. Yang *et al*., in a series of 15 cadaveric specimens, also observed that cases in which the AEA traveled apart from the skull base tended to exhibit more pneumatised ethmoid cells, although formal statistical testing was not performed.^19^

Developmentally, ethmoid sinuses are known to exist at birth as small cells and generally achieve adult size by approximately 12 years of age.^31^ Frontal sinus aeration begins postnatally and may progress into late adolescence or even early adulthood, approximately up to 20–25 years.^32^ This prolonged and individually variable pneumatisation of the ethmoid and frontal sinuses provides a plausible basis for the wide range of adult Keros types and AEA types. Paediatric CT studies have shown that age correlates positively with both H_LL_ and D_AS_,^33^^,^^34^ supporting that the extent of sinus pneumatisation might be associated with AEA position.

This study has limitations. Its retrospective nature introduces potential selection bias, and the sample size was modest. Given the 1-mm slice thickness of the PNS CT scans, slight measurement errors in anatomical lengths and angles are possible. Nonetheless, to the best of our knowledge, we provide a distinctive contribution by quantifying not only lateral lamella height and the presence of a SOEC but also frontal sinus volume and FRAC metrics to examine their relationships with AEA type. Taken together, larger V_FS_, larger FRAC, the presence of SOEC and a taller lateral lamella were each associated with a greater likelihood that the AEA courses farther from the skull base. These findings support a potential connection between the extent of frontal and ethmoidal pneumatisation and AEA configuration. Clarifying causality, however, will require more studies with standardised imaging and anatomical correlation.

- The anterior ethmoidal artery (AEA) shows variable relationships to the skull base, influencing surgical risk during endoscopic sinus surgery
- Using 3D-reconstructed paranasal sinus CT, this study quantified frontal and ethmoidal pneumatisation parameters to identify anatomical predictors of AEA configuration
- A free-hanging AEA was associated with greater frontal sinus and frontal recess anterior cell volumes, increased lateral lamella height and a higher prevalence of supraorbital ethmoid cells
- The AEA-skull base distance showed significantly positive correlations with lateral lamella height, frontal sinus volume and both the volume and height of frontal recess anterior cells

## Conclusion

In this retrospective study, larger frontal sinus volumes, increased FRAC dimensions or height, the presence of SOEC and a taller lateral lamella were each associated with an AEA course positioned apart from the skull base and/or an increased AEA–skull base distance. These observations indicate that the degree of pneumatisation in the frontal sinus and ethmoid cells might play a determining role in anatomical patterns of AEA.

## Data Availability

The datasets generated and/or analysed during the current study are available in the Zenodo repository [https://zenodo.org/records/18112611].
